# Study on the Mechanisms of Ischemic Stroke Impacting Sleep Homeostasis and Circadian Rhythms in Rats

**DOI:** 10.1111/cns.70153

**Published:** 2025-02-17

**Authors:** Ting‐ting Chu, Chen Sun, Yong‐hui Zheng, Wen‐ying Gao, Lin‐lin Zhao, Jing‐yu Zhang

**Affiliations:** ^1^ Department of Neurology Fourth Affiliated Hospital of Harbin Medical University Harbin China; ^2^ Department of General Surgery The 2nd Affiliated Hospital of Harbin Medical University Harbin China

**Keywords:** circadian rhythms, **c**ryptochrome 1 (Cry1), ischemic stroke, period 1 (Per1), sleep homeostasis

## Abstract

**Objective:**

This study aimed to investigate the impact of ischemic stroke (IS) on sleep homeostasis and circadian rhythms in rats, as well as the underlying mechanisms.

**Methods:**

The middle cerebral artery occlusion model was employed to induce IS in rats. Sixty young and sixty aged rats were randomly divided into six groups for experiments. Neurological function was assessed using the Garcia score, and infarct size was evaluated through 2,3,5‐triphenyltetrazolium chloride staining. Sleep–wake cycles were monitored by implanting electrodes into the neck muscles to record electroencephalograms and electromyograms. Parameters such as sleep latency, waking time, non‐rapid eye movement (NREM) sleeping, rapid eye movement sleeping, NREM delta power, and waking theta power were measured. Serum cortisol and melatonin levels were measured using enzyme‐linked immunosorbent assay. Gene and protein expression of circadian regulators period 1 (*Per1*) and cryptochrome 1 (*Cry1*) in the pineal gland were assessed using real‐time quantitative reverse transcription polymerase chain reaction and western blot.

**Results:**

Compared to the sham groups, IS‐induced rats showed a decrease in Garcia scores and an increase in cerebral infarction area. Besides, relative to young rats, aged rats exhibited more severe cerebral infraction damage, lower melatonin levels, higher cortisol levels, disrupted sleep–wake cycles, and altered gene and protein expression levels of *Per1* and *Cry1* in the pineal gland.

**Conclusions:**

IS can lead to neurological impairments and brain damage, with aged rats showing more severe effects. IS also disturbs melatonin and cortisol levels, affects sleep homeostasis, and results in disordered *Per1* and *Cry1* gene and protein expression levels. These findings underscore the role of circadian disruption and stress response in the pathology of IS, especially in aging populations.

## Graphical abstract

This research disclosed that ischemic stroke caused sensory motor deficits and different degrees of brain tissue damage in both young and aged rats, accompanied by disturbances in melatonin secretion, elevated cortisol levels, and disruptions in the sleep‐awakening cycle and the expression of circadian rhythm‐related genes Per1 and Cry1. In the future, further exploration of these issues and mechanisms will be conducted, thereby providing treatment ideas for related diseases.

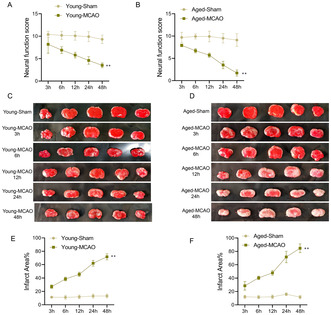

**Abbreviations**
AP: anterior–posterior
CCA: common carotid artery
Cry1: cryptochrome 1
ELISA: enzyme‐linked immunosorbent assay
ICA: internal carotid artery
IRE1: inositol‐requiring enzyme 1
IS: ischemic stroke
MCA: middle cerebral artery
NREM: non‐rapid eye movement
Per1: period 1
PERK: R‐like endoplasmic reticulum kinase
REM: rapid eye movement
RT‐qPCR: reverse transcription polymerase chain reaction
SPF: specific pathogen‐free
TTC: triphenyltetrazolium chloride

## 1 Introduction

The occurrence of ischemic stroke (IS) is primarily caused by the interruption or rupture of blood flow in arteries supplying either specific local regions or the overall region of the brain [1]. The pathogenesis of IS is complex, involving multiple biological processes such as neural damage, inflammatory response, and endocrine disorders [1]. Global studies have shown that the incidence of IS significantly increases with age. From 1990 to 2019, the incidence, mortality, and prevalence of stroke cases rose by 70.0%, 43.0%, and 102.0%, respectively [2]. Despite advancements in stroke management, recovery from IS is often complicated by secondary disorders, particularly sleep and circadian rhythm disruptions.

Emerging evidence suggests that IS patients frequently experience circadian rhythm alterations, largely due to disrupted cortisol and melatonin levels, which negatively affect recovery outcomes and quality of life. It has been reported that over 50% of stroke survivors develop sleep‐disordered breathing and other sleep disturbances [3, 4]. Melatonin, a neuroprotective hormone, plays a crucial role in maintaining circadian rhythms. Prior research has indicated that melatonin treatment before IS can mitigate neuronal damage by inhibiting endoplasmic reticulum stress and autophagy via the protein kinase R‐like endoplasmic reticulum kinase and inositol‐requiring enzyme 1 signaling pathways [5]. However, there is a lack of studies investigating the post‐stroke changes in melatonin levels and their specific impact on circadian disruptions.

Cortisol, a key hormone in the body's stress response, is secreted by the adrenal cortex and regulates various physiological processes, including energy metabolism, immune response, and cardiovascular function [6]. Acute cortisol elevation during IS is considered part of the body's protective response, aimed at stabilizing neurons and minimizing further damage. However, chronic or excessive cortisol elevation may result in a range of deleterious effects, such as immune suppression, heightened inflammation, and sleep disturbances [7, 8]. Despite its critical role, few studies have explored the long‐term effects of cortisol dysregulation on post‐stroke recovery, particularly concerning sleep–wake cycles and circadian rhythms.

The role of clock genes, such as period 1 (Per1) and cryptochrome 1 (Cry1), in regulating circadian rhythms and sleep–wake cycles is well established [9, 10]. However, the specific mechanisms by which ischemic events disrupt these genes and contribute to post‐stroke sleep disturbances remain poorly understood. Existing studies often fail to delve into the molecular changes that underlie circadian rhythm disruption following stroke. In addition, there is a scarcity of experimental data focusing on how IS influences both cortisol levels and clock gene expression simultaneously, particularly in relation to age‐related differences.

Therefore, the current research seeks to address these gaps by exploring the effects of stroke on sleep homeostasis, circadian rhythms, as well as melatonin and cortisol levels, while also examining the role of clock genes in mediating these changes. By focusing on both hormonal and genetic disruptions in IS, this study aimed to provide a more comprehensive understanding of the mechanisms underlying post‐stroke sleep disorders. Ultimately, this research was expected to lay the groundwork for novel therapeutic strategies targeting sleep and circadian disruptions, thereby improving recovery outcomes in stroke patients.

## 2 Materials and Methods

### 2.1 Experimental Animals

This study used 60 young Sprague–Dawley (SD) male rats (12 weeks old, 200–250 g) and 60 aged SD male rats (19–20 weeks old, 300–600 g). All rats were sourced from xxx and housed in a specific pathogen‐free animal facility. The environmental conditions were controlled at a temperature of 20°C–26°C, relative humidity of 40%–70%, and a 12‐h light/dark cycle. The animals were acclimatized for 1 week before the experiments. All procedures were approved by the Animal Ethics Committee of The 2nd Affiliated Hospital of Harbin Medical University (Approval No. SYDM2024‐087), and all experimental procedures followed the approved guidelines to ensure compliance with animal welfare regulations. Euthanasia was performed using an overdose of pentobarbital sodium (100 mg/kg intraperitoneally) to minimize animal suffering.

### 2.2 Instruments

For image processing, Fiji (a distribution of imageJ, 37370, UGO BASILE, Italy) was used. Brain tissue sections were prepared using an animal brain slicer (Zivic, Pittsburgh, PA, USA). Other instruments involved in this study were shown as follows: electrophoretic transfer system (#1704150, Trans‐Blot Turbo, Bio‐Rad, USA), universal high‐speed refrigerated centrifuge (Sorvall Biofuge Stratos, Thermo Scientific, USA), microplate reader (Spectra Max M4, USA), gel imaging system (Image Quant LAS4000, Germany GE Company).

### 2.3 Modeling and Grouping

The middle cerebral artery occlusion (MCAO) model was used to induce IS in rats. Rats were anesthetized with isoflurane (Sigma‐Aldrich, USA) and a 6‐0 silk suture was used to occlude the internal carotid artery and common carotid artery for 1 h, followed by reperfusion with phosphate‐buffered saline (PBS, pH = 7.4) (10010001, Thermo Fisher Scientific, USA). After reperfusion, neurological function and infarct area (IA) were assessed at various time points (3, 6, 12, 24, and 48 h) [11].

The experimental design included both young and aged rats, divided into the following groups (*n* = 10 per group), with the same procedure applied to both age groups: (1) Sham group: rats underwent surgery without suture insertion. (2) MCAO group: subjected to 1‐h ischemia followed by reperfusion. (3) Sham‐sleep deprivation group: underwent sleep deprivation during the last 6 h of the light phase. (4) MCAO‐sleep deprivation group: MCAO rats subjected to sleep deprivation during the last 6 h of the light phase. (5) Sham‐recovery group: rats underwent sleep deprivation and sleep recovery during the first 3 h of the dark phase. (6) MCAO‐recovery group: MCAO rats underwent sleep deprivation and sleep recovery during the first 3 h of the dark phase.

All procedures, including treatment and evaluation time points, were performed identically for both the young and aged rat groups, ensuring consistency in the experimental conditions across age groups.

### 2.4 Garcia Scoring for Neurological Function

The Garcia scoring system was used to assess motor deficits, including spontaneous activity, symmetry of limb movements, forelimb extension, climbing ability, proprioception, and whisker response. The total score ranged from 0 to 18, with lower scores indicating more severe motor deficit [12]. Assessments were performed in a quiet, dimly lit room (red light) during the last hour of the dark phase.

### 2.5 Tissue Pathology and 2,3,5‐Triphenyltetrazolium Chloride Staining

Rats were euthanized under isoflurane anesthesia by cervical dislocation. The brains were quickly removed, rinsed with PBS, and sectioned into 1 mm‐thick coronal slices. Sections were incubated with 0.05% 2,3,5‐triphenyltetrazolium chloride (TTC) solution at 37°C in the dark for 30 min, followed by PBS rinsing and storage in 10% buffered formalin. The levels of cerebral infarction in both groups of rats were observed at various time points, and the percentage of infarction was calculated. The IA was calculated by subtracting the healthy tissue area from the total contralateral hemisphere area. The percentage of infarction was expressed as the ratio of the infarcted area to the total hemisphere area.

### 2.6 Enzyme‐Linked Immunosorbent Assay

Serum levels of melatonin (#ab283259, Abcam, UK) and cortisol (#ab108665, Abcam, UK) were measured using enzyme‐linked immunosorbent assay (ELISA) kits according to the manufacturer's instructions. Blood samples in the young‐sham, young‐MCAO, aged‐sham, and aged‐MCAO groups were collected from the tail vein at 6, 12, 18, and 24 h post‐ischemia on days 2, 3, 5, and 7.

### 2.7 Quantitative Reverse Transcription Polymerase Chain Reaction Assay

Total RNA was extracted from pineal gland tissues using TRIzol reagent (Invitrogen, USA). RNA was reverse transcribed into complementary DNA using the reverse transcription kit (TAKARA, Japan). Quantitative reverse transcription polymerase chain reaction assay (qRT‐PCR) was performed using SYBR greenER qPCR SuperMix (TaKaRa, Japan) on a Roche LightCycler 96 system. The relative expression levels of Perl and Cry1 were normalized to GAPDH and calculated using the 2^−ΔΔCt^ method.

The primer sequences used were as follows: Perl (F, 5′‐CTCTCCGCAACCAGGATACC‐3′; R, 5′‐GCTAGGAGCTCTGAGAAGCG‐3′); Cry1 (F, 5′‐CTGAAGGAGTGCATCCAGGG‐3′; R, 5′‐TGTCCCCGGATCACAAACAG‐3′); GAPDH (F, 5′‐GAAGGTCGGTGTGAACGGAT‐3′; R, 5′‐GGGTTTCCCGTTGATGACCA‐3′).

### 2.8 Western Blot

The protein expression levels of Per1 and Cry1 in the pineal gland were measured at multiple time points (2nd, 3rd, 5th, and 7th days). The total protein was extracted using a protein extraction kit (Thermo Scientific Pierce, USA), and the protein concentration was measured through a bicinchoninic acid assay kit (Thermo Scientific, USA). Next, the protein samples were separated using sodium dodecyl sulfate‐polyacrylamide gel electrophoresis and transferred to the polyvinylidene fluoride (PVDF) membranes. The PVDF membranes were blocked with 5% non‐fat milk for 2 h. After blocking, the membranes were incubated with primary antibodies at 4°C overnight, followed by secondary antibodies conjugated with horseradish peroxidase for 2 h. Signals were detected using enhanced chemiluminescence and quantified using Image J. Primary antibodies used were as follows: Per1 (#13463‐1‐AP, 1:1000, Proteintech), Cry1 (#PA1‐527, 1:2000, Thermo Fisher Scientific), and β‐actin (#MA1‐140, 1:5000, Thermo Fisher Scientific). Secondary antibodies included goat anti‐rabbit (#A27034, 1:2000, Thermo Fisher Scientific) and goat anti‐mouse (#31430, 1:5000, Thermo Fisher Scientific).

### 2.9 Sleep‐Awake Cycle Monitoring

Electromyogram (EMG) electrodes were implanted above the neck muscles of rats to monitor sleep–wake cycles. After MCAO surgery, the rats were allowed to recover and adapt to the recording equipment for at least 5 days. During this period, continuous 48‐h EMG recordings were performed to verify the stability of electrophysiological signals and baseline sleep–wake cycles. Following a 12‐h recovery post‐surgery, sleep deprivation was performed during the last 6 h of the light phase, followed by a 3‐h recovery in the dark phase. EMG signals were recorded continuously throughout the light and dark periods.

### 2.10 Sleep Deprivation

Sleep deprivation was conducted on the second‐day post‐MCAO. Electroencephalography and EMG were recorded on days 2, 3, 5, and 7 to assess key sleep parameters, including waking time, non‐rapid eye movement (NREM) sleep time, rapid eye movement (REM) sleep time, NREM delta power, and REM theta power, across 24‐h cycles encompassing both light and dark periods. Circadian rhythms amplitude for wakefulness was calculated using the circadian index of wake (CI wake) formula: CI wake = (average dark—average light)/average 24 h.

Additionally, sleep latency, waking time, NREM and REM sleep, as well as NREM delta and REM theta power, were analyzed for the young‐sham‐sleep deprivation, young‐MCAO‐sleep deprivation, aged‐sham‐sleep deprivation, and aged‐MCAO‐sleep deprivation groups.

### 2.11 Statistical Analysis

Statistical analyses were conducted using SPSS 21 software and graphs were created with Graphpad software. The Shapiro–Wilk test was employed to assess data normality, and Levene's test was applied to check the homogeneity of variance. For normally distributed data, results were expressed as mean ± standard deviation, and one‐way analysis of variance (ANOVA) was used for multiple comparisons, while independent samples *t*‐tests were used for comparisons between two groups. For non‐normally distributed data, non‐parametric tests such as the Mann–Whitney *U* test and the Kruskal–Wallis test were applied. Additionally, Pearson correlation analysis was utilized to evaluate the relationship between neurological deficits and IA. *p* < 0.05 was considered statistically significant.

## 3 Results

### 3.1 IS Impairs Neural Function and Damages Brain Tissues

**FIGURE 1:**
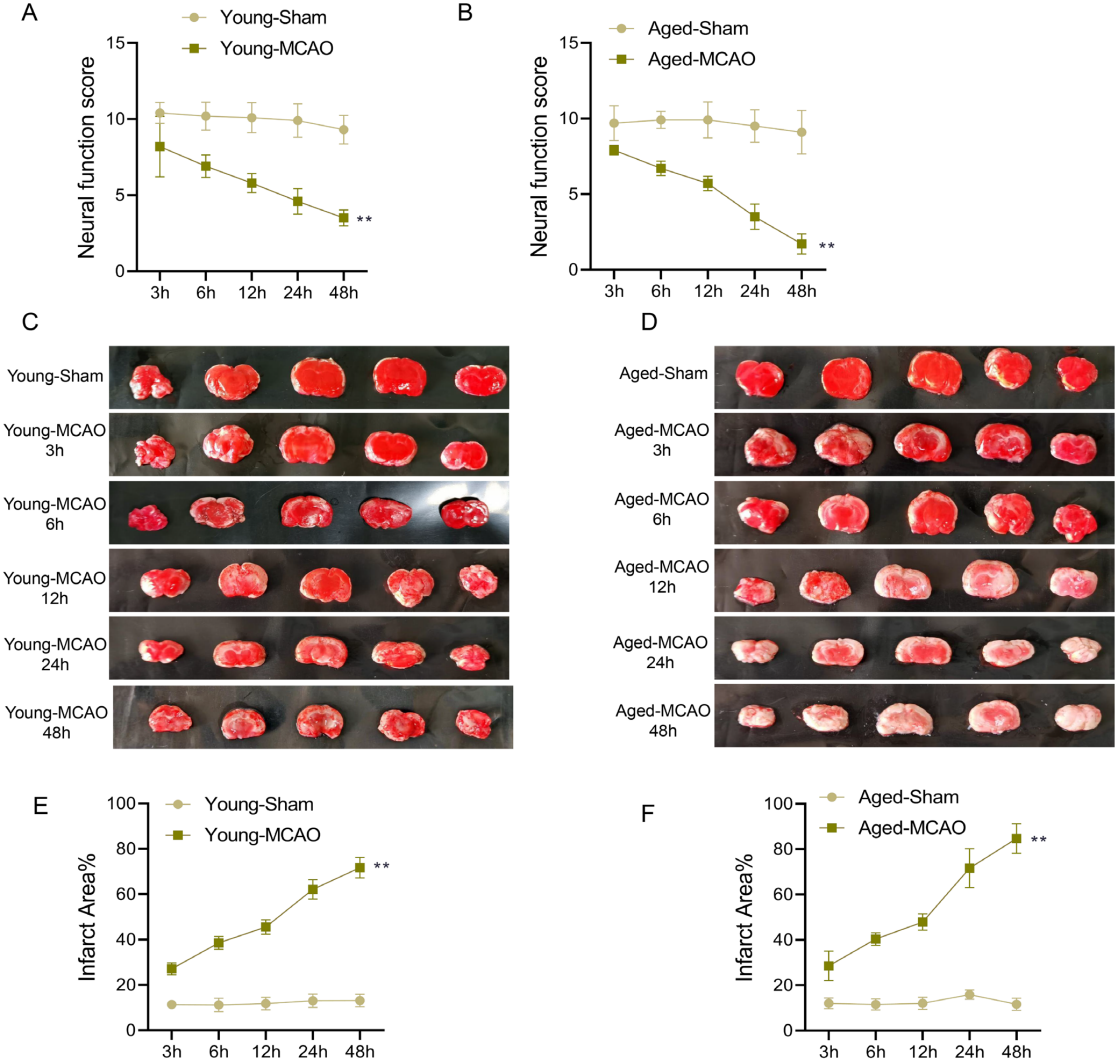
Evaluation of the ischemic stroke model in young and aged rat groups. (A, B) Garcia scores decline over time in young (A) and aged (B) rats post‐MCAO surgery; (C, D) Representative TTC‐stained images showing infarct morphology at different time points in young (D) and aged (E) rats; (E, F) Quantification of infarct area in young (E) and aged (F) rats. Data are presented as the mean ± standard deviation (*n* = 10); **p < 0.01 vs. young‐sham group or aged‐sham group.

To evaluate the neurological impact of IS, Garcia scores were assessed at various time points post‐MCAO (3rd, 6th, 12th, 24th, and 48th hour). A significant reduction in Garcia scores was observed in the young‐MCAO group compared to the young‐sham group, with the decline becoming more pronounced over time (*p* < 0.01) (Figure 1A). Similarly, the aged‐MCAO group exhibited significantly lower Garcia scores than the aged‐sham group at all time points (*p* < 0.01) (Figure 1B). By the 48th hour, the reduction in Garcia scores was more pronounced in the aged‐MCAO group compared to the young‐MCAO group, indicating more severe neurological deficits in aged rats. These findings suggest that the extent of neurological damage worsens over time post‐ischemia and that aged rats experience more severe deficits.

TTC staining was used to visualize brain infarcts. There were no infarcts in the young‐sham and aged‐sham groups, while significant infarcts were observed in both MCAO groups (Figure 1C). In the young‐MCAO group, IAs increased with reperfusion time, peaking at the 48th hour (*p* < 0.01) (Figure 1E). Similarly, the aged‐MCAO group exhibited a significant increase in infarcts size over time compared to the aged‐sham group (*p* < 0.01) (Figure 1D,F). These findings suggested that brain tissue damage progressively worsens post‐ischemia, with aged rats experiencing larger IAs than younger rats.

**FIGURE 2:**
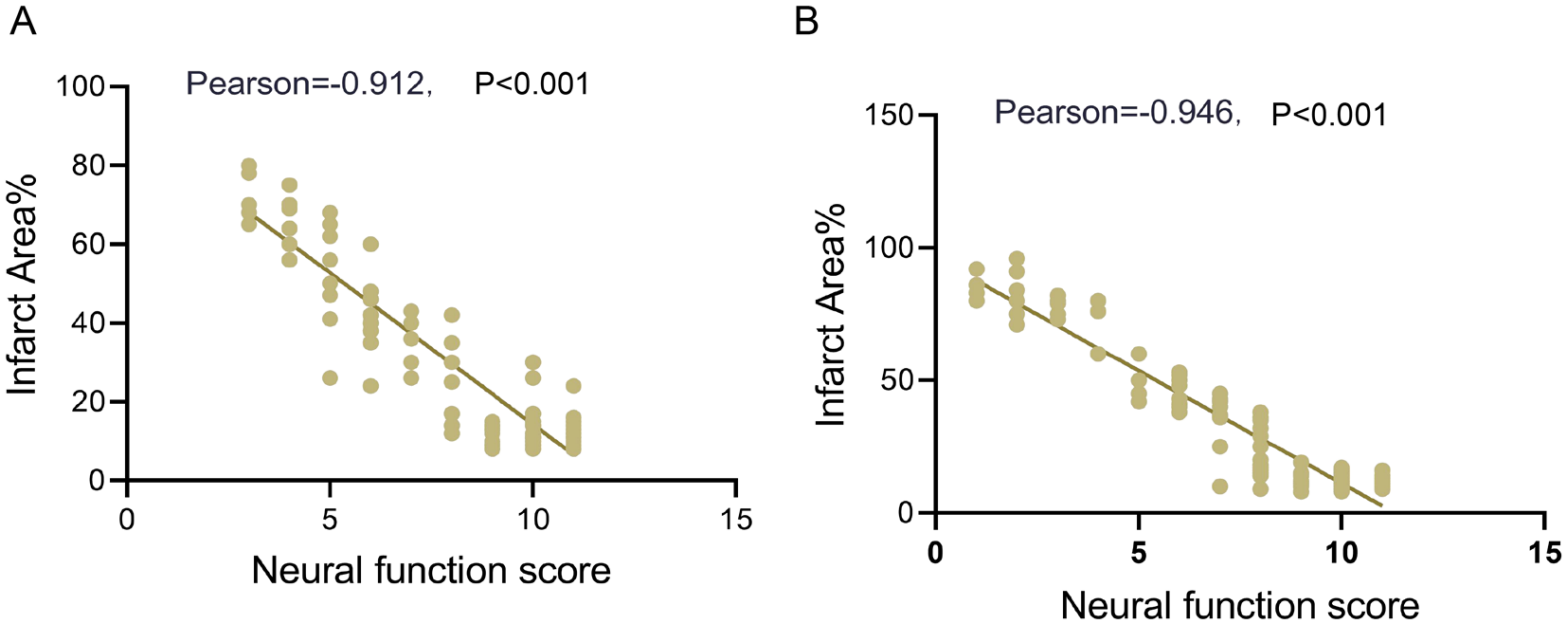
Correlation analysis between Garcia scores and infarct areas in young and aged rat group. (A) Pearson correlation analysis in the young rat group; (B) Pearson correlation analysis in the aged rat group.

Pearson correlation coefficient analysis revealed a strong negative correlation between Garcia scores and infarct percentage in both young (*r* = −0.912, *p* < 0.001) and aged rats (*r* = −0.946, *p* < 0.001) (Figure 2), indicating that larger IAs were associated with more severe neurological deficit. This suggested a direct relationship between brain infarction and functional impairment in IS rats.

### 3.2 IS Alters Serum Melatonin and Cortisol Levels

**FIGURE 3:**
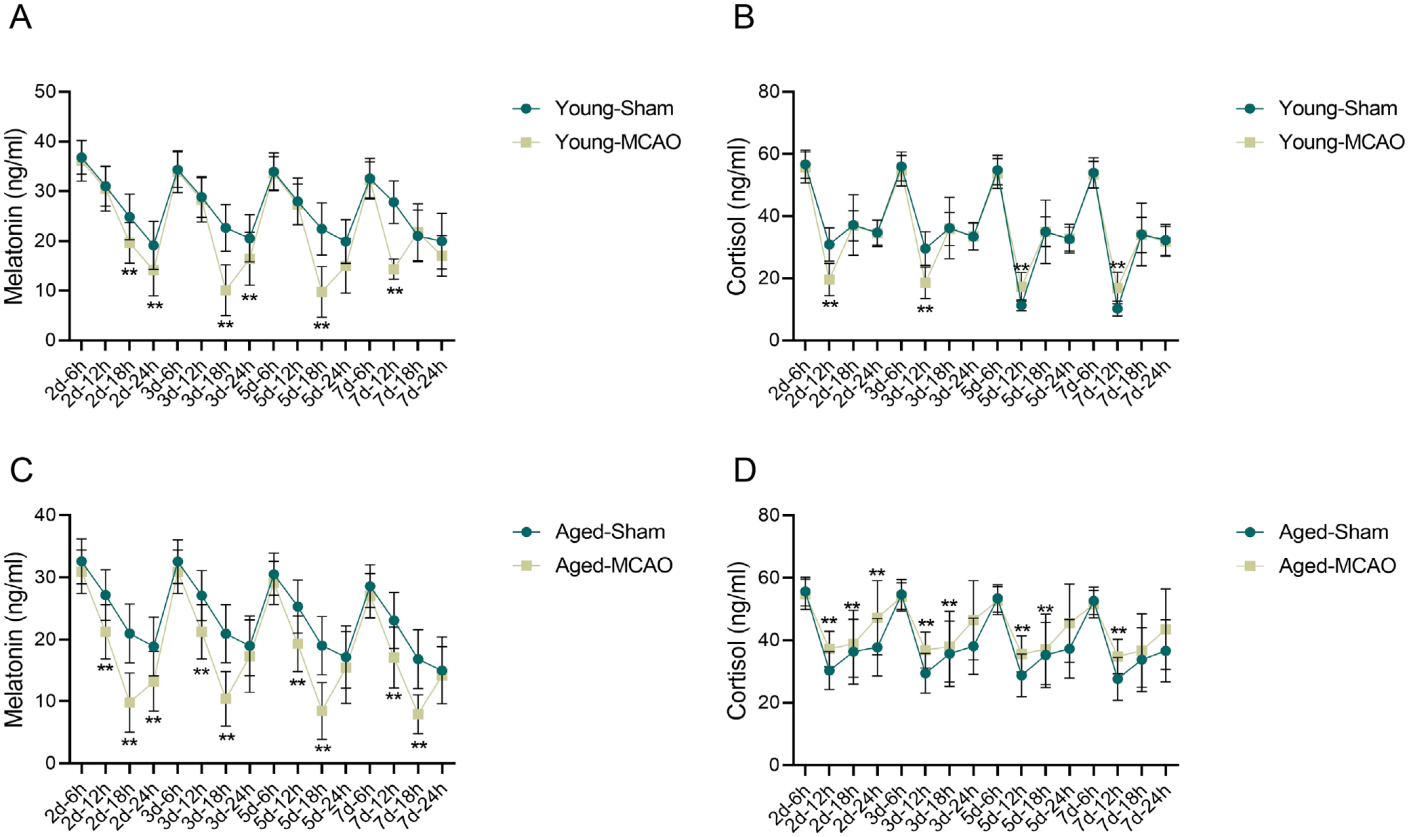
Changes in serum melatonin and cortisol levels in young and aged rats following ischemic stroke. (A) Serum melatonin levels in young rats; (B) Serum melatonin levels in aged rats; (C) Cortisol secretion levels in young rats; (D) Cortisol secretion levels in aged rats. Data are presented as the mean ± standard deviation (*n* = 10); **p < 0.01 vs. young‐sham group or aged‐sham group.

Changes in melatonin and cortisol levels in the serum of rats were detected using ELISA. The results showed that melatonin levels in the young‐MCAO group were significantly lower than in the young‐sham group at multiple time points (2d‐18 h, 2d‐24 h, 3d‐18 h, 3d‐24 h, 5d‐18 h, and 7d‐12 h) (*p* < 0.01), with the most notable reductions occurring in the evening hours (Figure 3A). In the aged rats, the aged‐MCAO group had significantly lower melatonin levels than the aged‐sham group at nearly all time points (*p* < 0.01), with the most pronounced reduction observed at 18 h on days 2, 3, 5, and 7 (Figure 3B).

Cortisol levels were also assessed, revealing a complex pattern. In the young‐MCAO group, cortisol levels were lower than in the young‐sham group at 2d‐12 h and 3d‐12 h but were higher at 5d‐12 h and 7d‐12 h (*p* < 0.01) (Figure 3C). In the aged rats, the aged‐MCAO group showed consistently higher cortisol levels than the aged‐sham group at all time points, particularly during the night and morning (*p* < 0.01) (Figure 3D).

These results indicated that IS caused a substantial disruption in melatonin levels and cortisol rhythms, with aged rats showing more pronounced and severe disturbances.

### 3.3 Sleep–Wake Cycle Disruptions in IS Model Rats

#### 3.3.1 Analysis of 24‐h Sleep–Wake Cycle Changes

**FIGURE 4:**
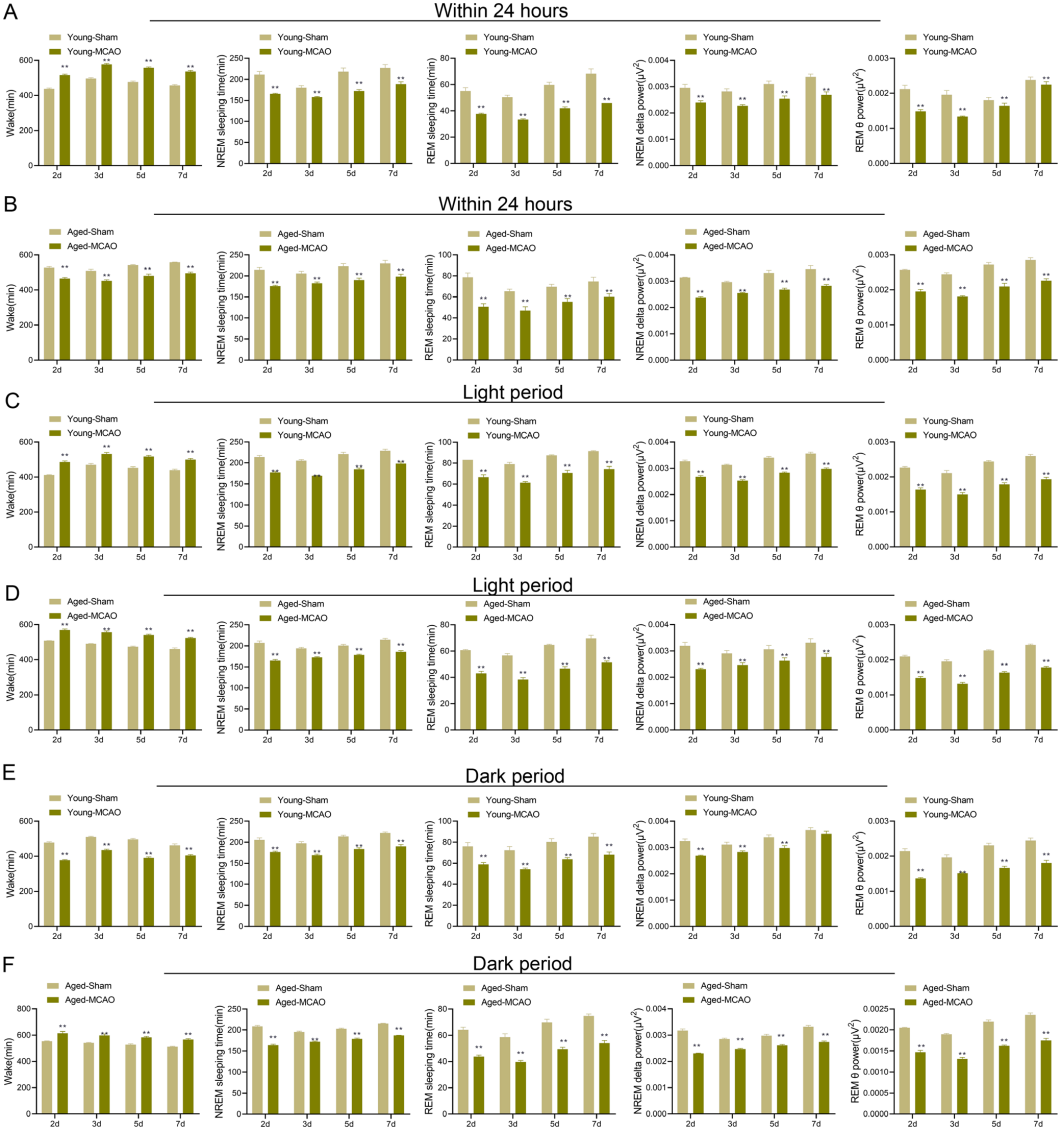
Sleep–wake cycle changes in young and aged rats after ischemic stroke at days 2, 3, 5, and 7. (A) Changes in wake, NREM sleep, REM sleep, NREM delta power, and REM theta power in the young‐sham group and young‐MCAO group during the 24‐h period; (B) Changes in the same indices in the aged‐sham group and aged‐MCAO group during the 24‐h period; (C) Changes in the same indices in the young‐sham and young‐MCAO groups during the light period; (D) Changes in the same indices in the aged‐sham and aged‐MCAO groups during the light period; (E) Changes in the same indices in the young‐sham and young‐MCAO groups during the dark period; (F) Changes in the same indices in the aged‐sham and aged‐MCAO groups during the dark period. Data are presented as the mean ± standard deviation (*n* = 3); **p < 0.01 vs. young‐sham group or aged‐sham group.

To assess how IS affects the sleep–wakening cycle, we monitored sleep–wake patterns over 24 h on days 2, 3, 5, and 7 post‐MCAO. The results showed that compared to the rats of the young‐sham group, the young‐MCAO group had a significant increase in waking time (*p* < 0.01), while a decrease in NREM, REM, NREM Delt, and REM theta sleep (*p* < 0.01) (Figure 4A). Similarly, the aged‐MCAO group displayed significantly decreased waking time and reduced NREM, REM, NREM Delt, and REM theta sleep compared to the aged‐sham group (*p* < 0.01) (Figure 4B). These results suggested that IS profoundly disrupted the sleep–wake cycle in both young and aged rats, with marked reductions in the time spent in different stages of sleep.

#### 3.3.2 Analysis of Sleep–Wake Cycles During the Light Phase

The effects of IS on sleep during the light phase were also analyzed. Compared to the young‐sham group, the young‐MCAO group showed a significant increase in waking time and a reduction in NREM, REM, NREM Delt, and REM theta sleep during the light phase (*p* < 0.01) (Figure 4C). Aged‐MCAO rats exhibited similar sleep–wake cycle disruptions during the light period, with increased waking time and reduced sleep (*p* < 0.01) (Figure 4D). These findings highlighted the severity of stroke‐induced disruptions in the sleep–wake cycle, especially during the light period when sleep was expected to dominate.

#### 3.3.3 Analysis of Sleep–Wake Cycles During the Dark Phase

During the dark phase, the young‐MCAO group exhibited decreased waking time. NREM, REM, NREM Delt, and REM theta sleep compared to the young‐sham group (Figure 4E). However, in aged rats, the aged‐MCAO group showed a significant increase in waking time and a significant decrease in all sleep parameters compared to the aged‐sham group (*p* < 0.01) (Figure 4F). The results indicated that stroke impacted sleep differently during the dark phase for young and aged rats, with aged rats showing a more profound disruption.

### 3.4 Effects of Sleep Deprivation on the Sleep–Wake Cycle of IS Rats

**FIGURE 5:**
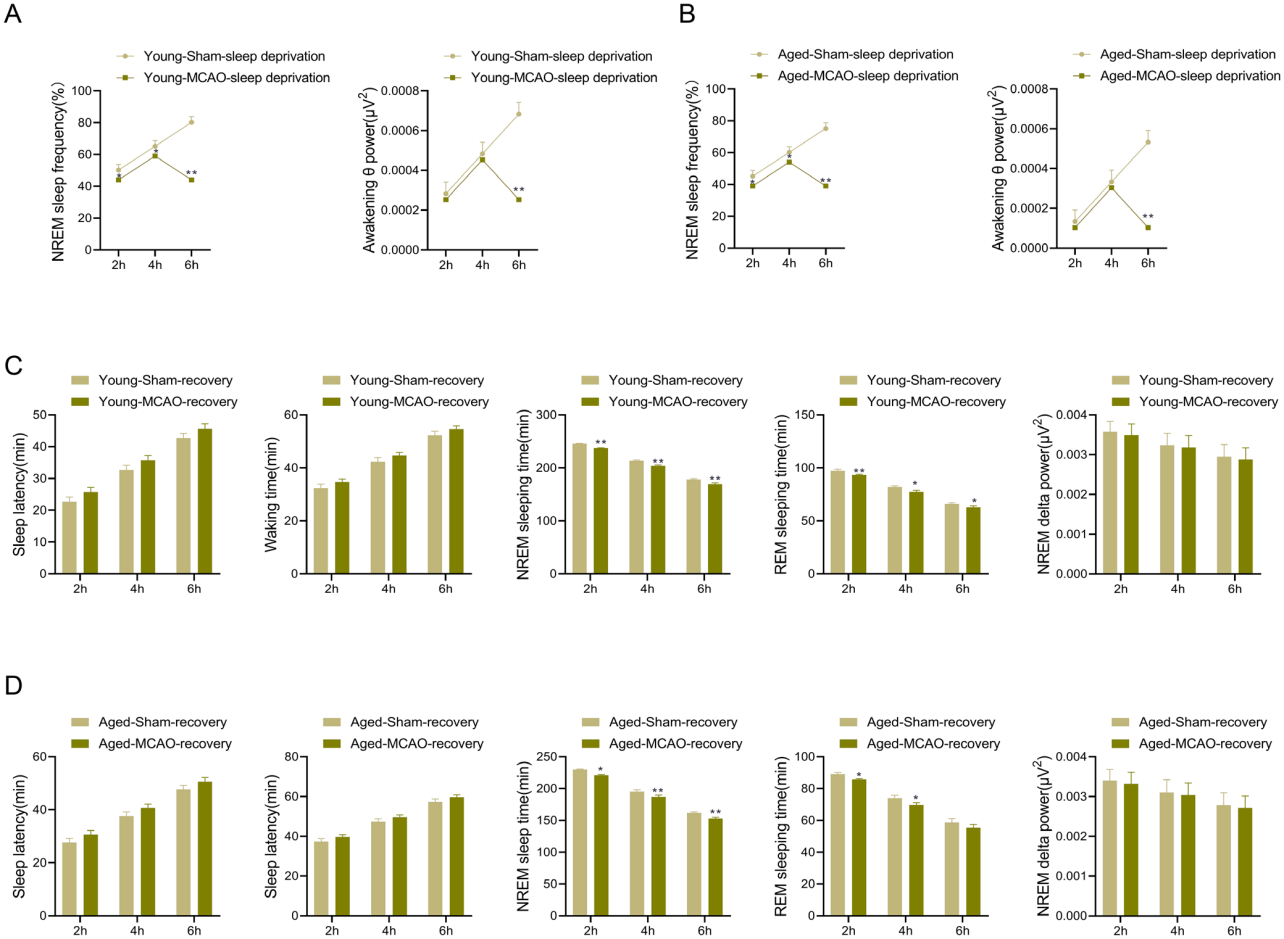
Effects of sleep deprivation and recovery on the sleep–wake cycle in young and aged ischemic stroke model rats. (A, B) Analysis of NREM sleep frequency and waking delta power during sleep deprivation in young (A) and aged (B) rats; (C, D) Sleep latency, waking time, NREM, REM, and NREM delta during the recovery period in young (C) and aged (D) rats. Data are presented as the mean ± standard deviation (*n* = 3); *p < 0.05, **p < 0.01 vs. young‐sham‐sleep recovery group or aged‐sham‐sleep recovery group.

To assess the impact of sleep deprivation on IS recovery, rats were subjected to sleep deprivation, and NREM sleep frequency and theta power were observed and recorded at the 2nd, 4th, and 6th hour. The results showed that compared to the rats in the young‐sham‐sleep deprivation group, NREM sleep frequency and theta power significantly decreased at the 6th hour in the young‐MCAO‐sleep deprivation group (*p* < 0.01) (Figure 5A). A similar trend was observed in the aged rats (*p* < 0.01) (Figure 5B). These results suggest that sleep deprivation exacerbates sleep disturbances in IS rats, further impairing the sleep–wake cycle.

### 3.5 Sleep Recovery Analysis

Following sleep deprivation, rats were allowed a recovery period, and sleep parameters were recorded at the 2nd, 4th, and 6th hours. In the young‐MCAO‐recovery group, sleep latency and waking time slightly increased, while NREM and REM sleep significantly decreased compared to the young‐sham‐recovery group (*p* < 0.05) (Figure 5C). Similarly, compared to the aged‐sham‐recovery group, the aged‐MCAO‐recovery group also showed a slight increase in sleep latency and waking time, with NREM and REM showing varying degrees of reduction (Figure 5D). These findings indicated that IS impaired the ability to recover sleep after deprivation.

### 3.6 Changes in mRNA Levels of Per1 and Cry1 in the Pineal Gland

**FIGURE 6:**
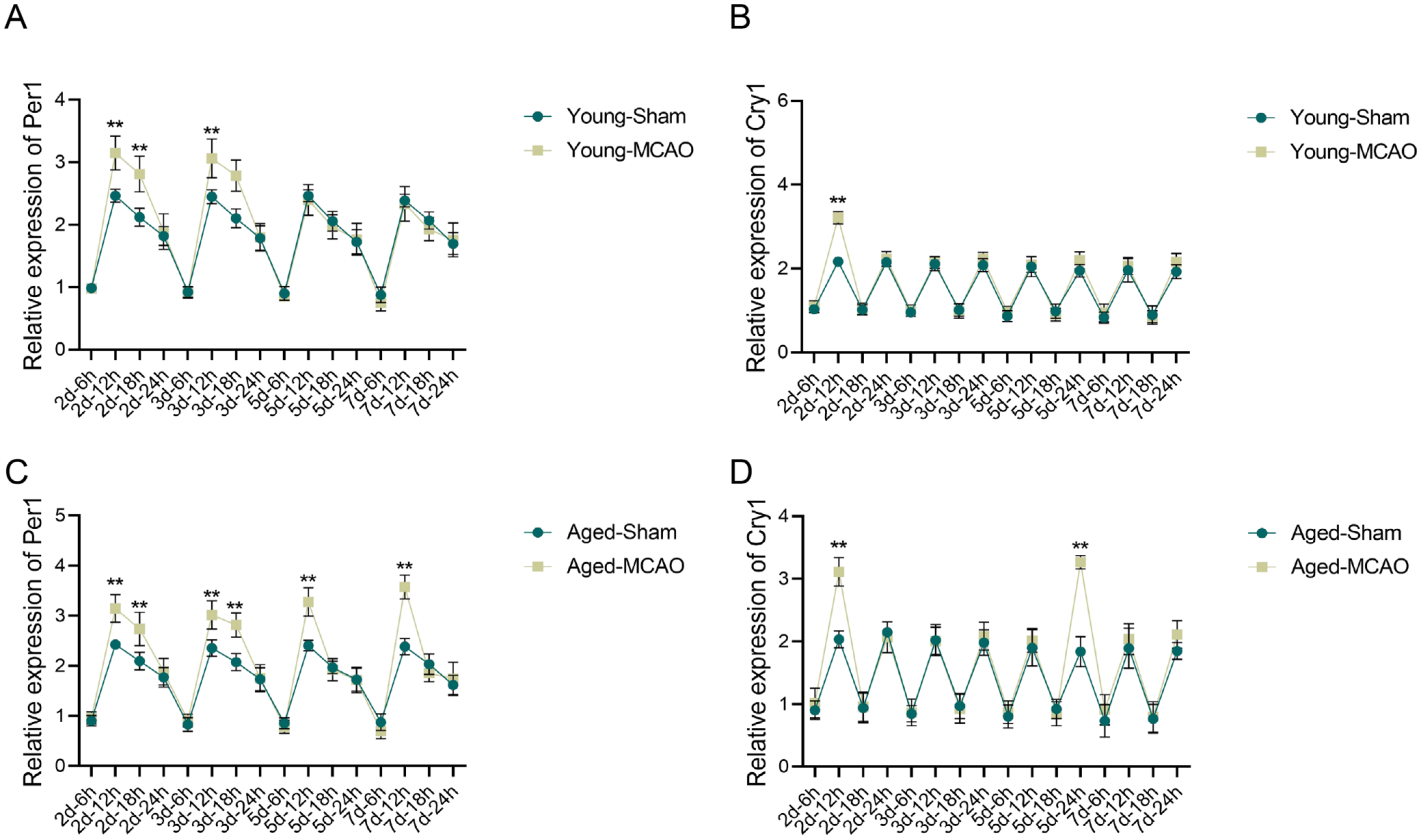
mRNA expression changes of *Per1* and *Cry1* in the pineal gland in the young and aged rats on days 2, 3, 5, and 7 post‐ischemic stroke. (A, B) Relative mRNA expression levels of *Per1* (A) and *Cry1* (B) in young rats; (C, D) Relative mRNA expression levels of *Per1* (C) and *Cry1* (D) in aged rats. Data are presented as the mean ± standard deviation (*n* = 3); **p < 0.01 vs. young‐sham group or aged‐sham group.

RT‐qPCR was used to detect the mRNA expression of *Per1* and *Cry1* in the pineal gland at days 2, 3, 5, and 7 post‐MCAO. In the young‐MCAO group, *Per1* expression was significantly elevated at 12th and 18th on days 2 and 3 compared to the young‐sham group (*p* < 0.01) (Figure 6A). Besides, the mRNA expression of *Cry1* was significantly elevated at 12th on day 2 (*p* < 0.01) (Figure 6B). The aged‐MCAO group also showed significant increases in *Per1* expression at multiple time points (2d‐12 h, 2d‐18 h, 3d‐12 h, 3d‐18 h, 5d‐12 h, and 7d‐12 h) (*p* < 0.01) (Figure 6C), with *Cry1* levels elevated at 2d‐12 h and 5d‐24 h (*p* < 0.01) (Figure 6D). The above results indicated that the IS altered the expression of key circadian genes, contributing to disruptions in circadian rhythms.

### 3.7 Changes in Protein Expression of Per1 and Cry1 in the Pineal Gland

**FIGURE 7:**
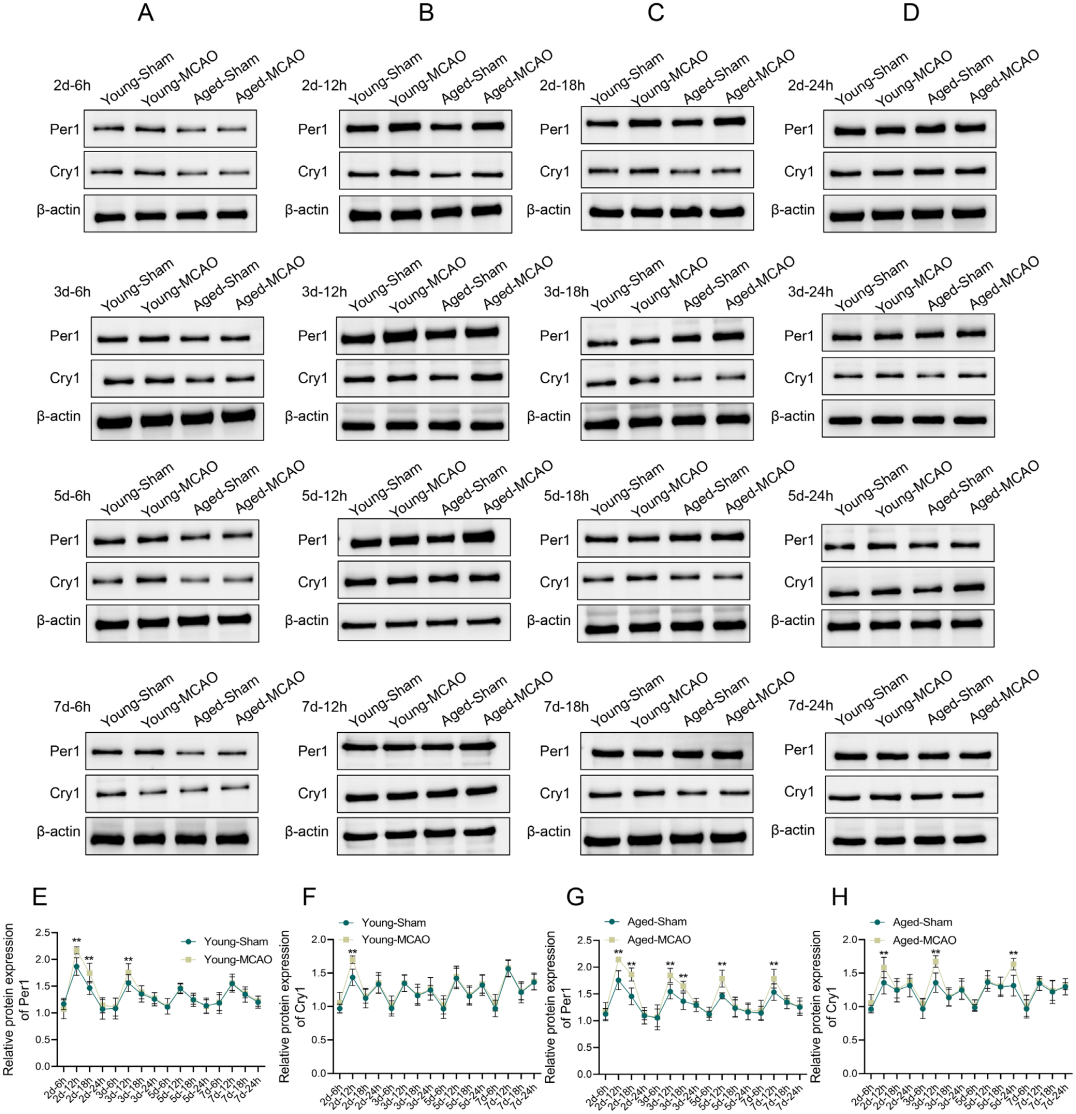
Protein expression changes of *Per1* and *Cry1* in the pineal gland of young and aged rats at days 2, 3, 5, and 7 after ischemic stroke. (A) Representative protein bands for *Per1* and *Cry1* at 6, 12, 18, and 24 h on day 2; (B) Representative protein bands for *Per1* and *Cry1* at 6, 12, 18, and 24 h on day 3; (C) Representative protein bands for *Per1* and *Cry1* at 6, 12, 18, and 24 h on day 5; (D) Representative protein bands for *Per1* and *Cry1* at 6, 12, 18, and 24 h on day 7; (E) Quantification of *Per1* protein expression in young rats; (F) Quantification of *Cry1* protein expression in young rats; (G) Quantification of *Per1* protein expression in aged rats; (H) Quantification of *Cry1* protein expression in aged rats. Data are presented as the mean ± standard deviation (*n* = 3); **p < 0.01 vs. young‐sham group or aged‐sham group.

As shown in Figure 7, western blot results revealed that *Per1* protein levels were significantly elevated at 2d‐12 h, 2d‐18 h, and 3d‐12 h (*p* < 0.01), and *Cry1* levels were significantly elevated at 12th hour on day 2 (*p* < 0.01). Similar increases were observed in aged rats, with *Per1* (2d‐12 h, 2d‐18 h, 3d‐12 h, 3d‐18 h, 5d‐12 h, and 7d‐12 h) and *Cry1* (2d‐12 h, 3d‐12 h, and 5d‐24 h) protein levels significantly elevated at multiple time points (*p* < 0.01). These results indicated that IS altered the expression of key circadian proteins, which likely contributed to the observed disruptions in the sleep–wake cycle.

## 4 Discussion

In this study, we utilized the Garcia score to assess the neural function of rats in the IS model at multiple time points [13]. The results demonstrated a gradual decrease in Garcia scores over time, indicating worsening neurological deficits. Furthermore, TTC staining revealed a negative correlation between the Garcia scores and infarct size, confirming that the MCAO model effectively induced IS, resulting in varying degrees of cerebral infarction and neurological impairment. The successful construction of this model in rats provides a reliable basis for studying the pathogenesis of IS and potential therapeutic interventions.

Our findings demonstrated significant disruption of the circadian rhythm and sleep–wake cycle in all groups following MCAO surgery. These results were consistent with previous research, which have shown that stroke can severely impact circadian regulation and sleep homeostasis. Circadian rhythms are fundamental for maintaining physiological balance, regulating metabolism, endocrine function, and cardiovascular health, as well as sleep–wake cycles. Disruption of the circadian clock can lead to various complications, including sleep disorders [14]. Clinically, monitoring the sleep–wake cycle in stroke patients can provide valuable insights into their condition and aid in prognosis and recovery strategies.

Previous studies have also highlighted the link between stroke and melatonin rhythm disruption [14]. Melatonin secretion rhythms is a reliable marker of circadian rhythms, and alterations in its levels have been associated with sleep disturbances in stroke patients [15, 16]. In our study, we observed a significant reduction in melatonin levels in IS rats, consistent with previous research showing that melatonin plays a protective role in reducing infarct size and preserving neural function after stroke by modulating inflammatory and oxidative stress signaling pathways [17]. The reduced melatonin levels observed in our IS model likely contribute to circadian rhythm disruption and worsened infarct outcomes. These findings suggest that supplementing melatonin in clinical settings could help mitigate post‐stroke sleep disorders and enhance recovery.

In addition to melatonin, cortisol levels were significantly elevated in IS rats, and this elevation correlated with the severity of the stroke [18]. Elevated cortisol levels are known to be part of the body's natural stress response aimed at maintaining neuronal stability. However, chronic elevation of cortisol can have detrimental effects, including immune suppression, metabolic disturbances, and further disruption of sleep patterns. Our results corroborate previous findings, showing that cortisol levels increase after stroke, which may be a compensatory mechanism to protect neural cells. However, prolonged cortisol elevation can lead to adverse consequences, such as immune suppression, metabolic disorders, and sleep deprivation [19, 20]. Therefore, controlling cortisol levels through anti‐inflammatory or anti‐stress medications may offer a viable therapeutic strategy to improve post‐stroke outcomes [21, 22, 23].

Our study also revealed increased expression of the clock genes *Per1* and *Cry1* in the IS model at specific time points. These genes play crucial roles in regulating the biological clock and maintaining circadian rhythms [24, 25, 26]. Alterations in their expression can lead to sleep–wake cycle disruptions. For example, previous studies have revealed that *Per1* and *Per2* mRNA expression increases following sleep deprivation, which may further impact circadian regulation [14, 17]. *Per1* has also been implicated in cellular reprogramming and the modulation of inflammatory responses [27], while *Cry1* variants have been associated with delayed sleep phase disorders [28] and cell proliferation regulation via hypoxia‐inducible factor signaling [29]. In our study, the observed increases in *Per1* and *Cry1* expression suggest that these genes may play a key role in mediating the circadian disturbances seen after stroke. Further exploration of these molecular pathways could provide insights into the development of therapeutic interventions targeting circadian rhythm restoration in stroke patients.

Although our study sheds light on the impact of IS on sleep–wake cycles, circadian rhythms, and the differential expression of *Per1* and *Cry1*, several limitations must be acknowledged. First, the experimental design is relatively simple, and the mechanisms underlying the observed gene expression changes were not fully elucidated. Moreover, the data supporting the specific regulatory pathways of melatonin and cortisol in IS remain insufficient. Future studies should incorporate more in‐depth mechanistic analyses, such as investigating the role of inflammatory pathways and oxidative stress in modulating clock gene expression. In addition, extending the research to include long‐term follow‐up and therapeutic interventions will provide a more comprehensive understanding of how sleep and circadian disruptions can be managed in clinical settings. Ultimately, while this study provides a foundation for exploring the role of circadian regulation in stroke recovery, further research is necessary to refine these findings and translate them into clinical applications.

## 5 Conclusion

In conclusion, this research disclosed that IS caused sensory motor deficits and different degrees of brain tissue damage in both young and aged rats, accompanied by marked disturbances in melatonin secretion, elevated cortisol levels, and disruptions in the sleep–wake cycle, as well as altered expression of key circadian rhythm‐related genes *Per1* and *Cry1*. These findings highlight the role of circadian regulation in IS recovery, suggesting potential therapeutic targets. Future research will focus on elucidating the underlying mechanisms to inform new treatment strategies for post‐stroke sleep disorders and recovery.

## Author Contributions

T.‐t.C. performed the experiment and wrote the manuscript. C.S. and Y.‐h.Z. participated in the in vivo experiments. W.‐y.G. and L.‐l.Z. analyzed the data. J.‐y.Z. conceived the project and revised the manuscript.

## Ethics Statement

All experimental procedures involving animals were conducted in accordance with the ethical standards and guidelines for the care and use of laboratory animals and were approved by the Ethics Committee of The 2nd Affiliated Hospital of Harbin Medical University (Approval No. SYDM2024‐087).

## Consent

The authors have nothing to report.

## Conflicts of Interest

The authors declare no conflicts of interest.

## Supporting information


Data S1.

## Data Availability

The datasets used and/or analyzed during the current study are available from the corresponding author on reasonable request.
