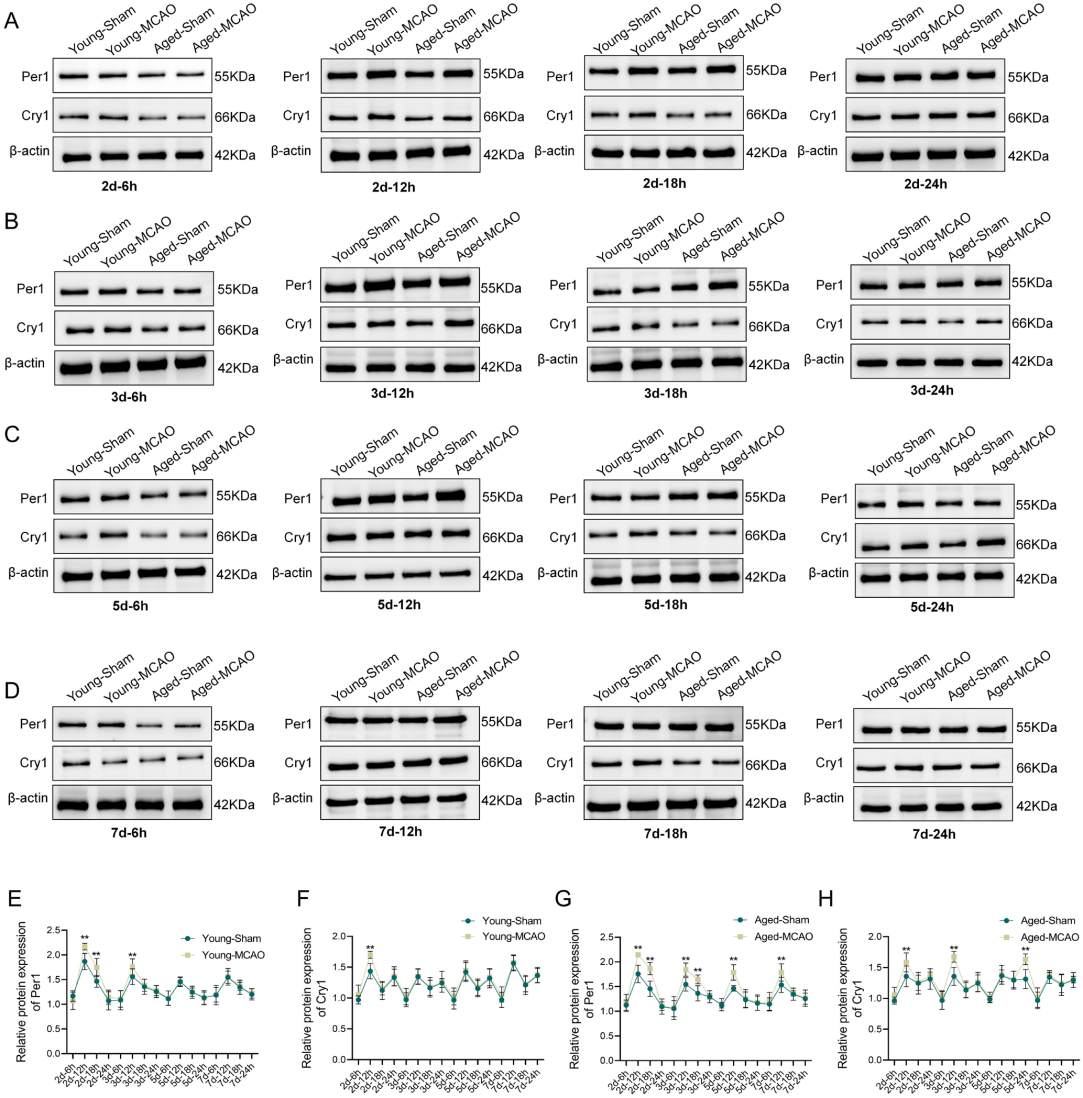# Correction to “Study on the Mechanisms of Ischemic Stroke Impacting Sleep Homeostasis and Circadian Rhythms in Rats”

**DOI:** 10.1002/cns.70776

**Published:** 2026-02-13

T. Chu, C. Sun, Y. Zheng, et al. “Study on the Mechanisms of Ischemic Stroke Impacting Sleep Homeostasis and Circadian Rhythms in Rats.” *CNS Neuroscience & Therapeutics* 31, no. 2 (2025): e70153. https://doi.org/10.1111/cns.70153.

The author identified a duplication error in Figure 7A, where the Western blot bands for Per1(2d‐6h) and Cry1(2d‐6h) were mistakenly replicated during image compilation. We wish to emphasize that the original experimental data remain intact and verifiable. This error does not impact the quantitative analysis, results, or conclusions of the study. The corrected Figure 7A is provided below.

We apologize for the error.