# Supplementary material for: Study on the Mechanisms of Ischemic Stroke Impacting Sleep Homeostasis and Circadian Rhythms in Rats
Source: CNS Neurosci Ther. 2025 Feb 17;31(2):e70153. doi: 10.1111/cns.70153 (PMC11831068; doi:10.1111/cns.70153)

Full unedited gel/blot for Figure 7A

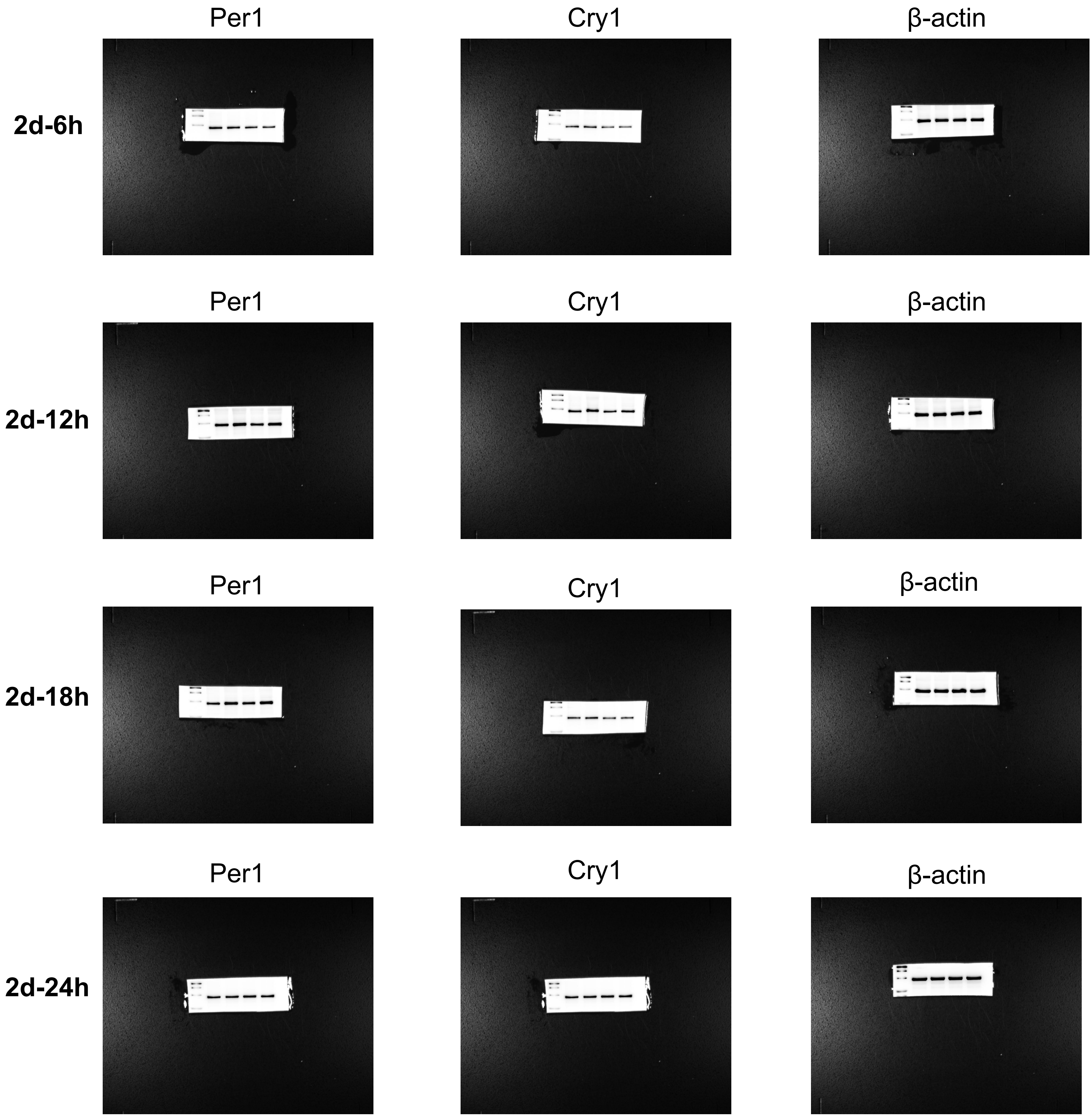

Full unedited gel/blot for Figure 7B

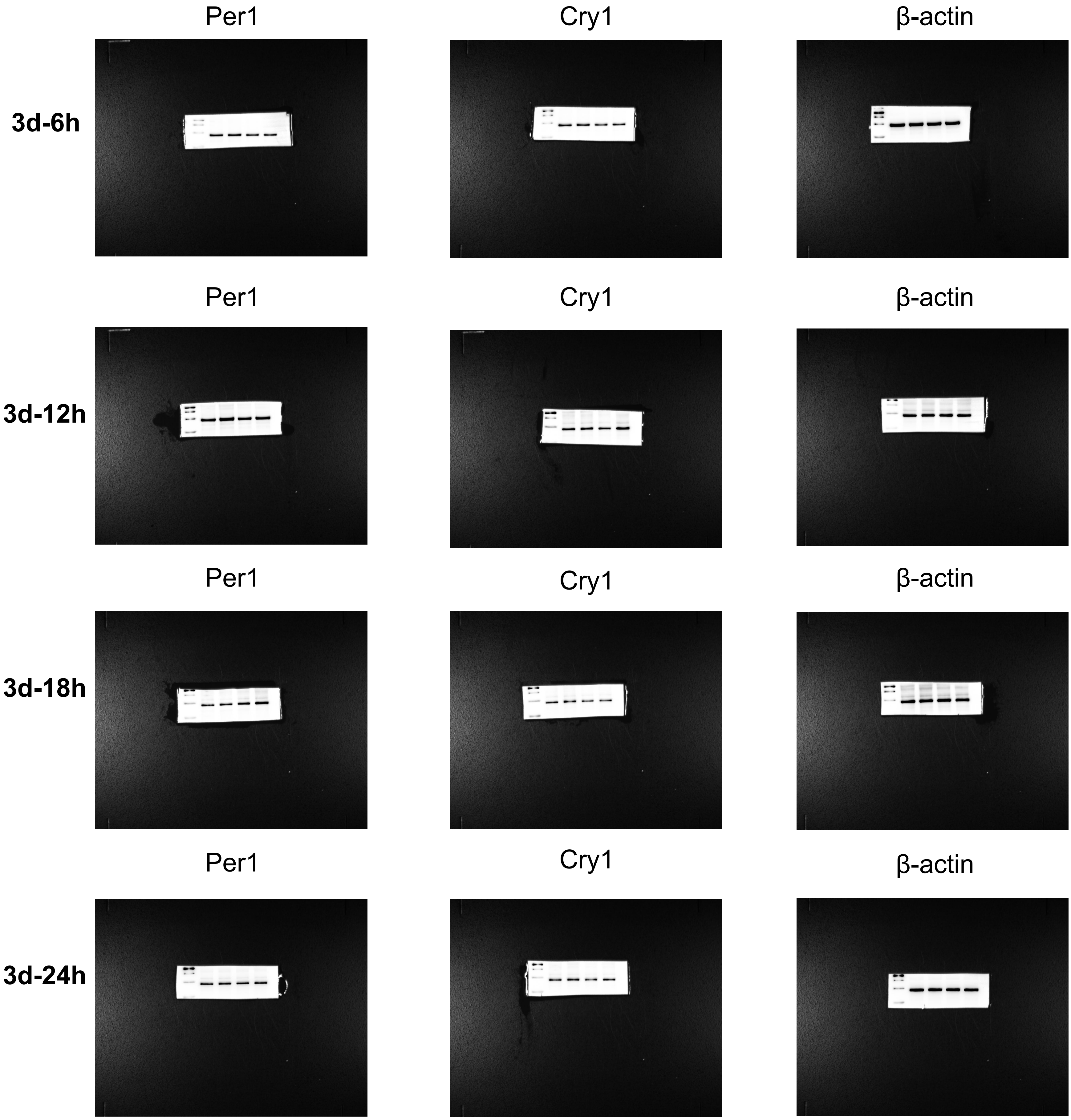

Full unedited gel/blot for Figure 7C

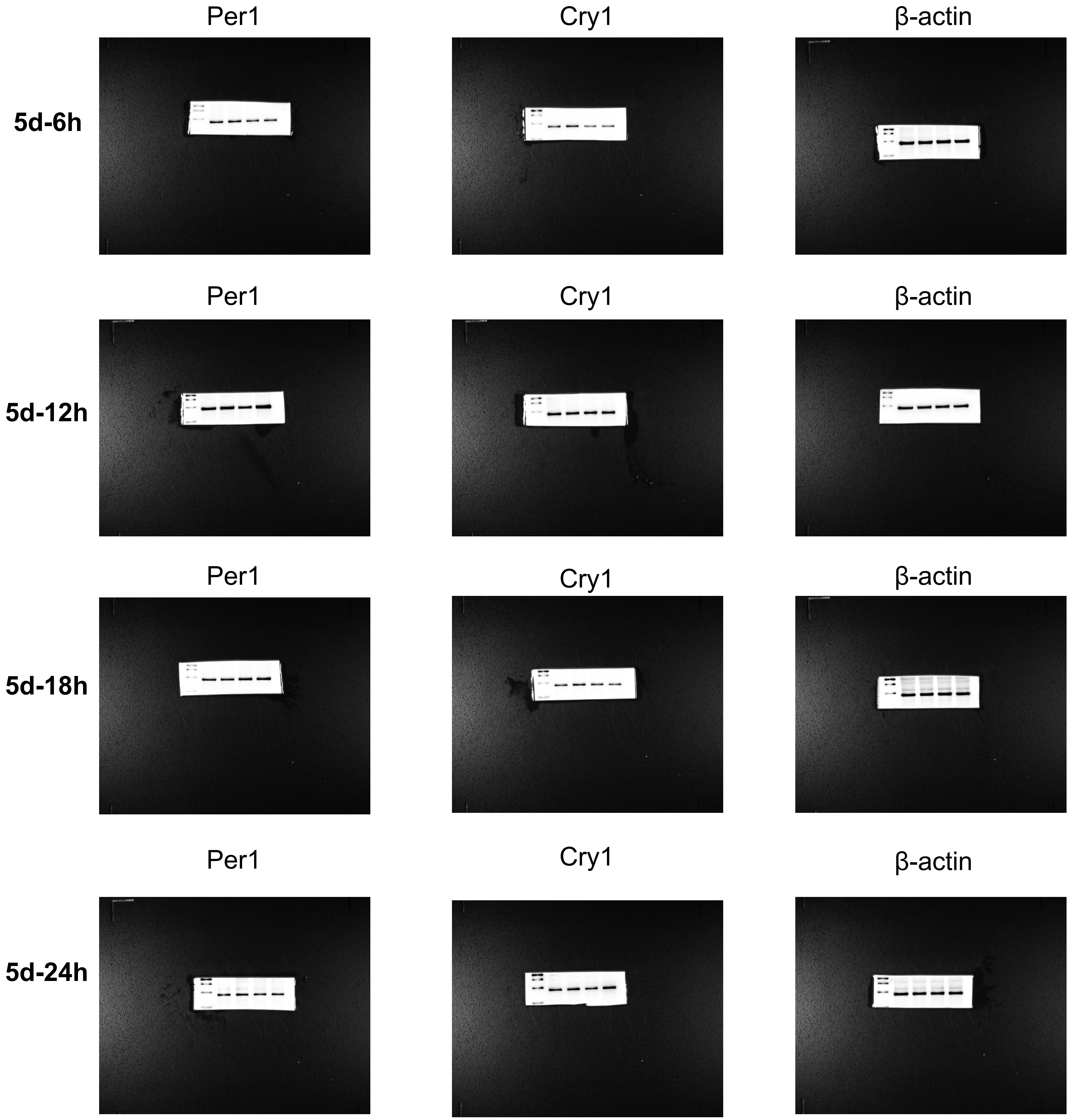

Full unedited gel/blot for Figure 7D

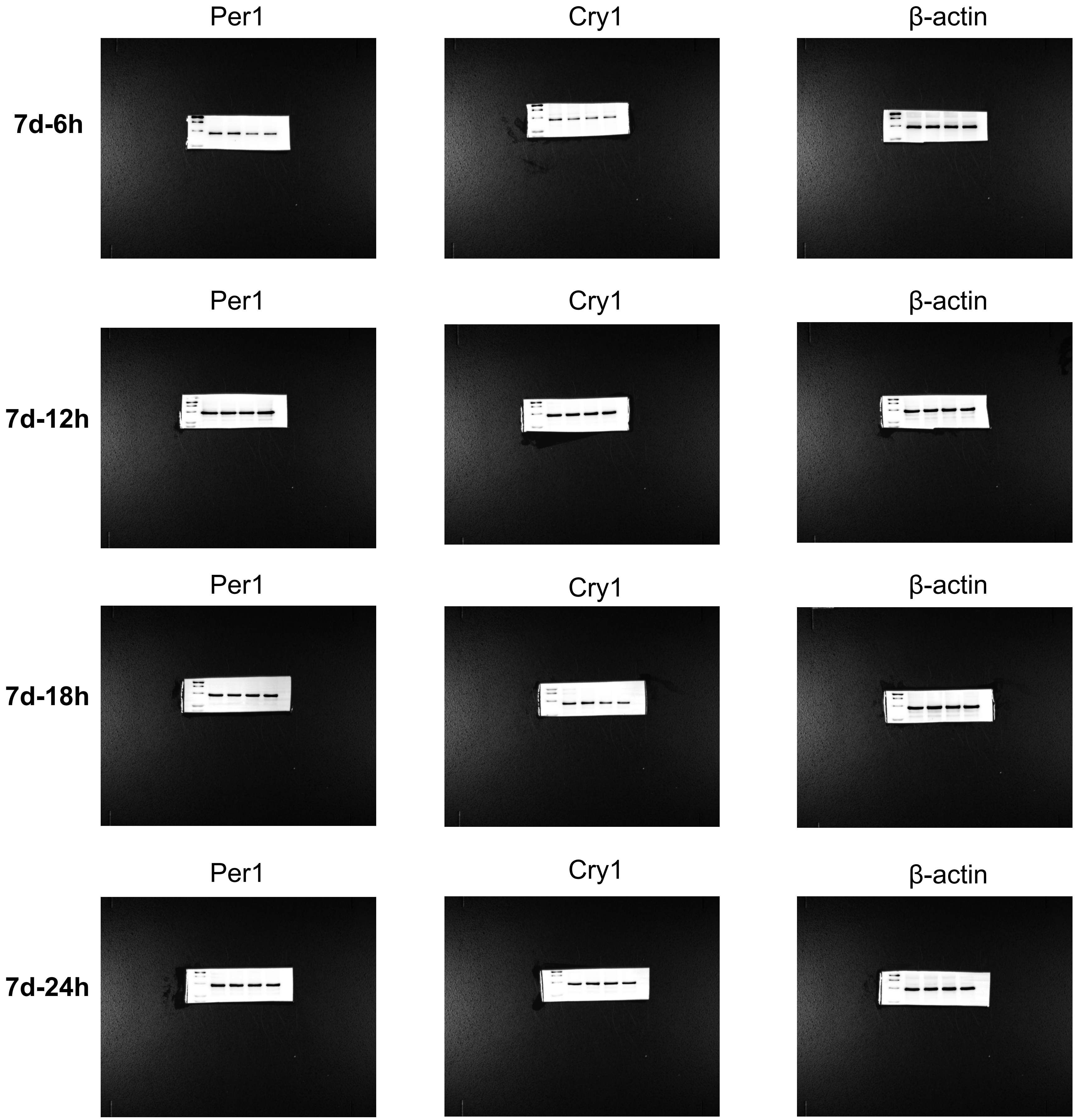

Supplement: Supplementary file 1 — Data S1. [file CNS-31-e70153-s001.pdf]
